# Temporal Relationship Between Activity Engagement and Cognition

**DOI:** 10.1093/geroni/igaa057.1987

**Published:** 2020-12-16

**Authors:** Seojung Jung, Karen Siedlecki

**Affiliations:** 1 SUNY College at Old Westbury, Old Westbury, New York, United States; 2 Fordham University, New York, New York, United States

## Abstract

Previous studies have shown that activity engagement is related to cognitive function. However, few studies have examined the temporal order between activity engagement and various domains of cognition. Using data from the Virginia Cognitive Aging Project (baseline N =5430, Mage =51.28, SD =18.12), we examined the temporal relationships between engagement in physical and cognitive activity and different cognitive domains (reasoning, spatial visualization, episodic memory, processing speed, vocabulary) after controlling for age, education, self-rated health and depression. Cross-lagged panel analyses indicate that very few of the temporal relationships between activity level and cognition were significant except higher levels of cognitive activity significantly predicted better future processing speed, but not the reverse. Findings suggest the importance of engaging in cognitively stimulating activities, which help adults preserve processing speed over time. This study also highlights the importance of longitudinal design on various domains of cognition to help develop domain-specific interventions.

